# Supplementary material for: Intragenic recruitment of NF-κB drives splicing modifications upon activation by the oncogene Tax of HTLV-1
Source: Nat Commun. 2020 Jun 16;11:3045. doi: 10.1038/s41467-020-16853-x (PMC7298006; doi:10.1038/s41467-020-16853-x)
Supplement: Supplementary file 1 — Supplementary Information [file 41467_2020_16853_MOESM1_ESM.pdf]

Supplementary figures

Intragenic recruitment of NF-kB drives splicing modifications upon activation by the oncogene Tax of HTLV-1

Ben Ameur *et al.*

Contents

Supplementary Figure 1-6

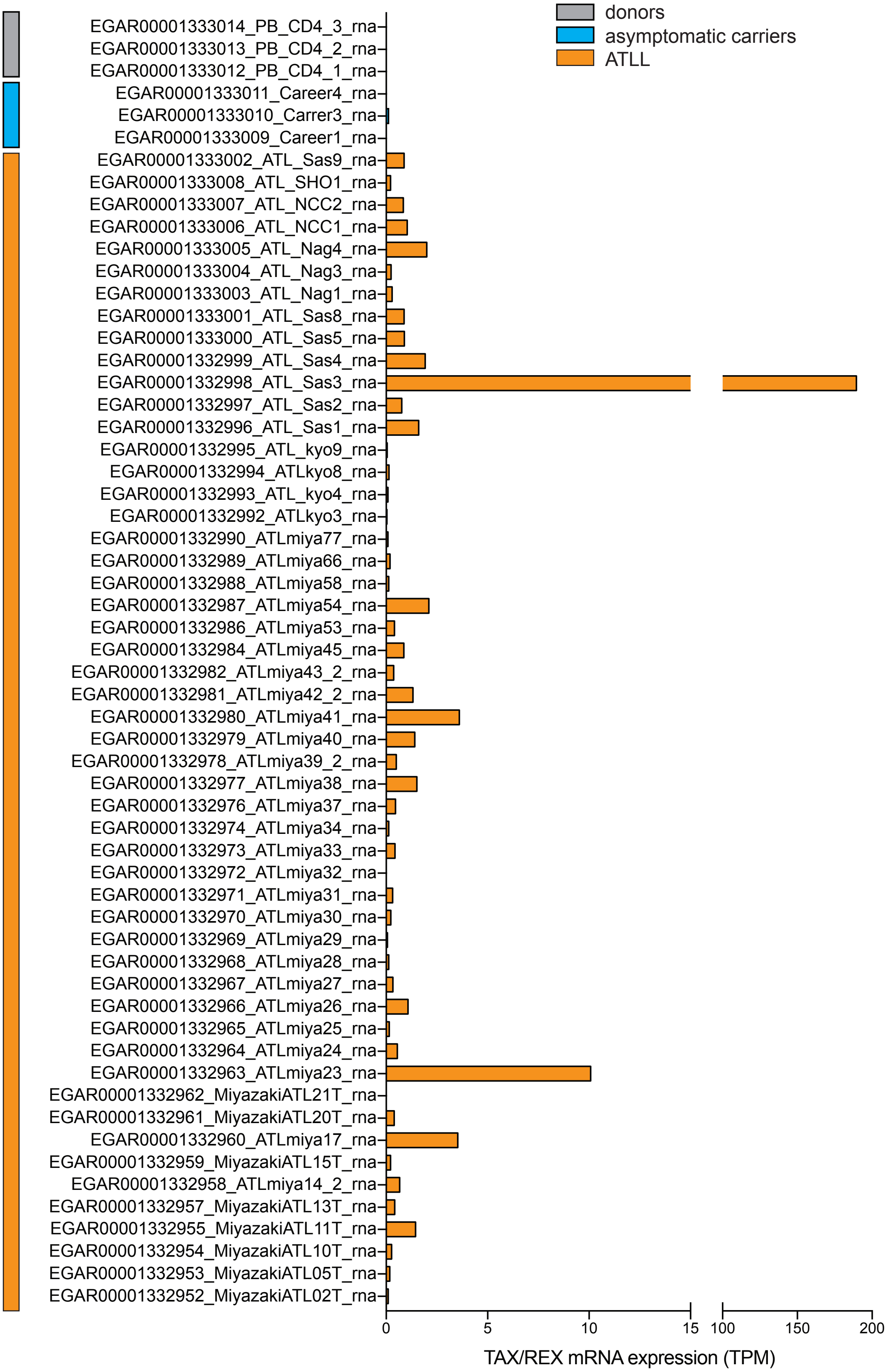

Supplementary Fig. 1: Abundance of TAX/REX transcripts in asymptomatic carriers and ATLL.

Kallisto[58] was used to quantify the abundance of TAX/REX mRNA (TPM, transcript per million), referring to the nucleotide sequence of NC_001436.1 (coordinates 6951-8078). Source data are provided as a Source Data file.

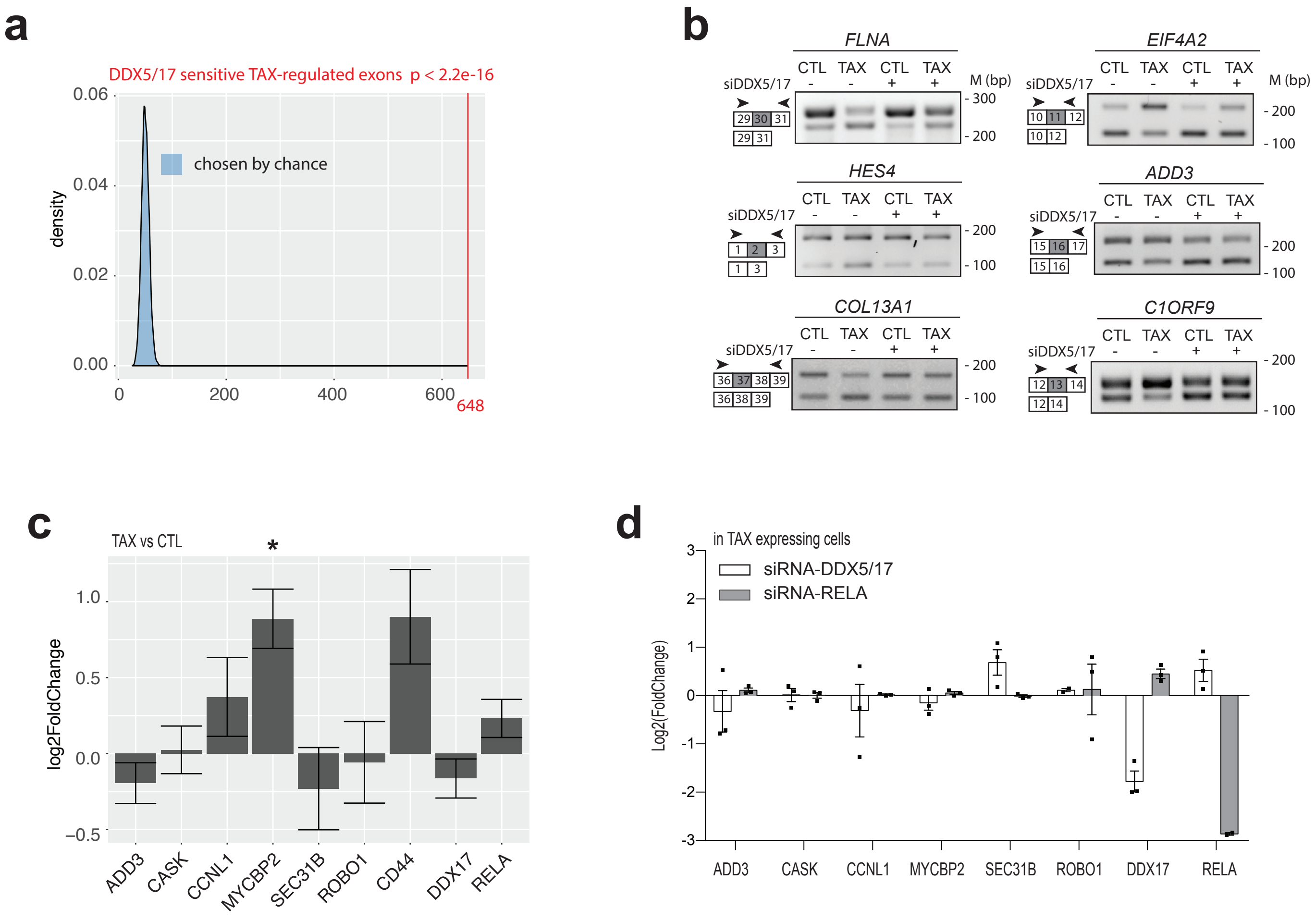

Supplementary Fig. 2: DDX5/17 expression regulates Tax splicing targets.

a Bootstrapped distribution of DDX5/17 sensitive Tax-regulated exons and 648 randomly chosen exons (10,000 repetitions) among overall expressed exons in HEK cells (blue). Using a bootstrap analysis, random samples were compared with the set of exons regulated by Tax and DDX5/17, and the normal distribution of these counts was used in a sample t-test to assess the significance of the exon events responsive to Tax and DDX5/17 expressions. One-sided sample t-test; $p = 2.2e^{-16}$ was considered significant. **b** Validation of RNA-seq data in DDX5/17-depleted cells using exon specific RT-PCR. Representative image from three independent experiments is shown. **c** Fold-change in gene expression of Tax splicing targets upon Tax expression. Values were obtained from DESeq2 analysis of RNA-seq data and expressed as log2(FoldChange). Statistical significance was determined with GLM and Wald test (MYCBP2, *pvalue = 0,0003). **d** Fold-change in gene expression of Tax splicing targets upon knockdown of DDX5/17 and RELA expression in Tax expressing cells. Histograms represent means ± SEM of three independent experiments. Source data are provided as a Source Data file.

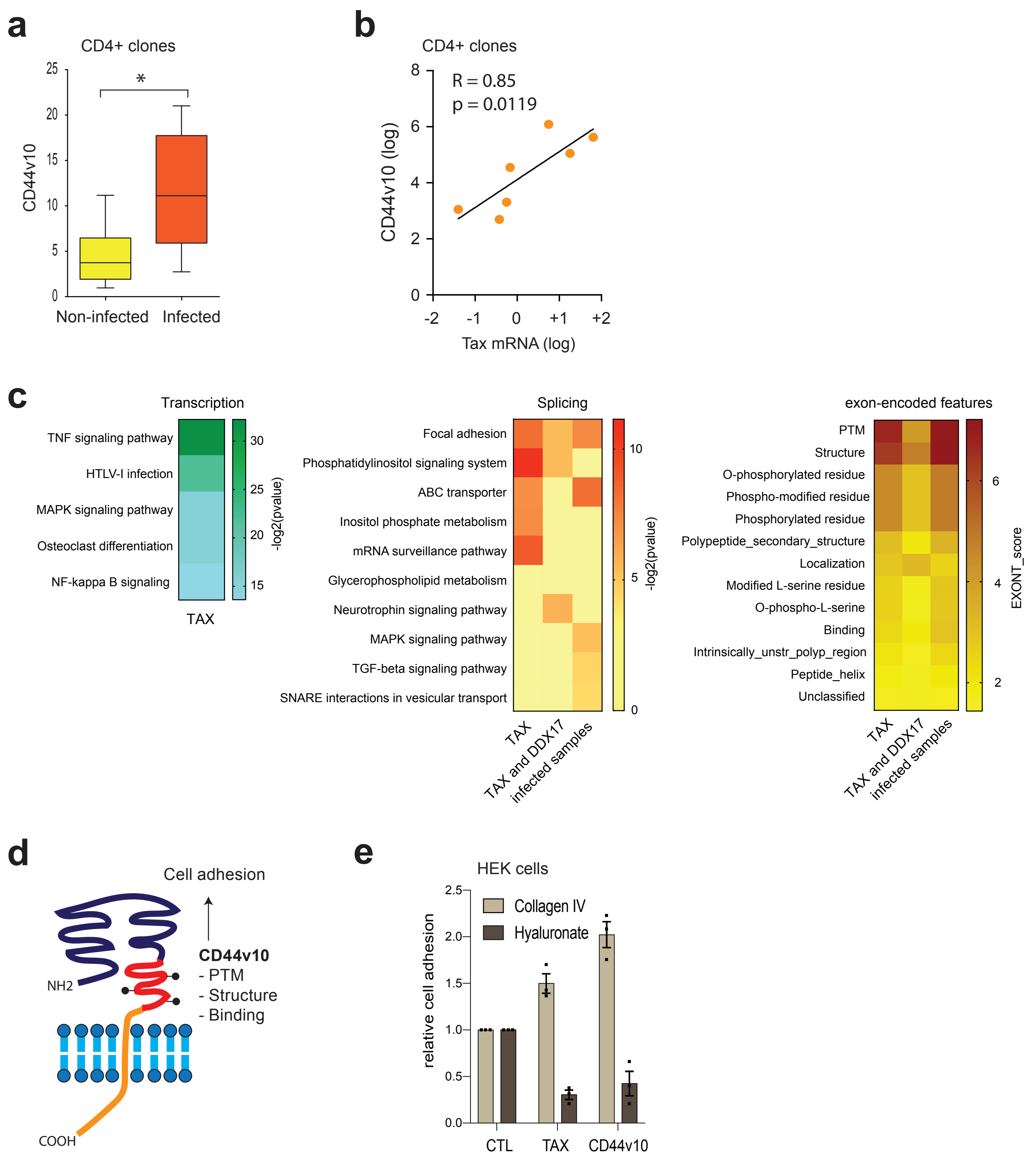

Supplementary Fig. 3: Functional insight to Tax-induced alternative splicing

a Expression of the splicing variant CD44v10 in HTLV-1 positive and negative cellular clones T-CD4+ derived from HAM/TSP patients (7 cellular clones in each category). CD44v10 mRNA was quantified by qRT-PCR from total RNA extracts. Median ± s.d. for non-infected vs infected clones were 11.1 ± 6.87 vs 3.7 ± 3.57; Mann-Whitney test, p = 0.0325. Boxes extend from the 25th to 75th percentiles, the mid line represents the median and the whiskers indicate the maximum and the minimum values. **b** Positive correlation between Tax and CD44v10 mRNA expression in seven infected CD4+ T-cell clones (Pearson correlation test). **c** Gene ontology (GO) analysis (DAVID) of Tax splicing and transcriptional targets. For splicing targets, GO and exon ontology (EO) analyses were carried out using genes and exon-cassette regulated by Tax or Tax and DDX17, or detected further in RNA-seq datasets (infected samples). Supplementary Table 2 presents the complete output of the EO analysis. The EXONT score represents the number of nucleotides covered by hits of the EXONT term divided by the total number of nucleotides of all the exons from the tested list[37]. **d** The CD44 protein structure. The red line represents alternative region encoded by variable exons v1-v10. The variable region encoded by exon v10 is a site of post-translational modifications (PTM) and protein interactions that contribute to cell adhesion properties of CD44. **e** Cell adhesion properties of HEK cells transiently transfected with control vector, pSG5M-Tax and pCEP4-CD44V10 on plate surfaces coated with Hyaluronic acid and type IV Collagen. Data are presented as the mean ± SEM values from biological replicates. Each black square represents a biological replicate.

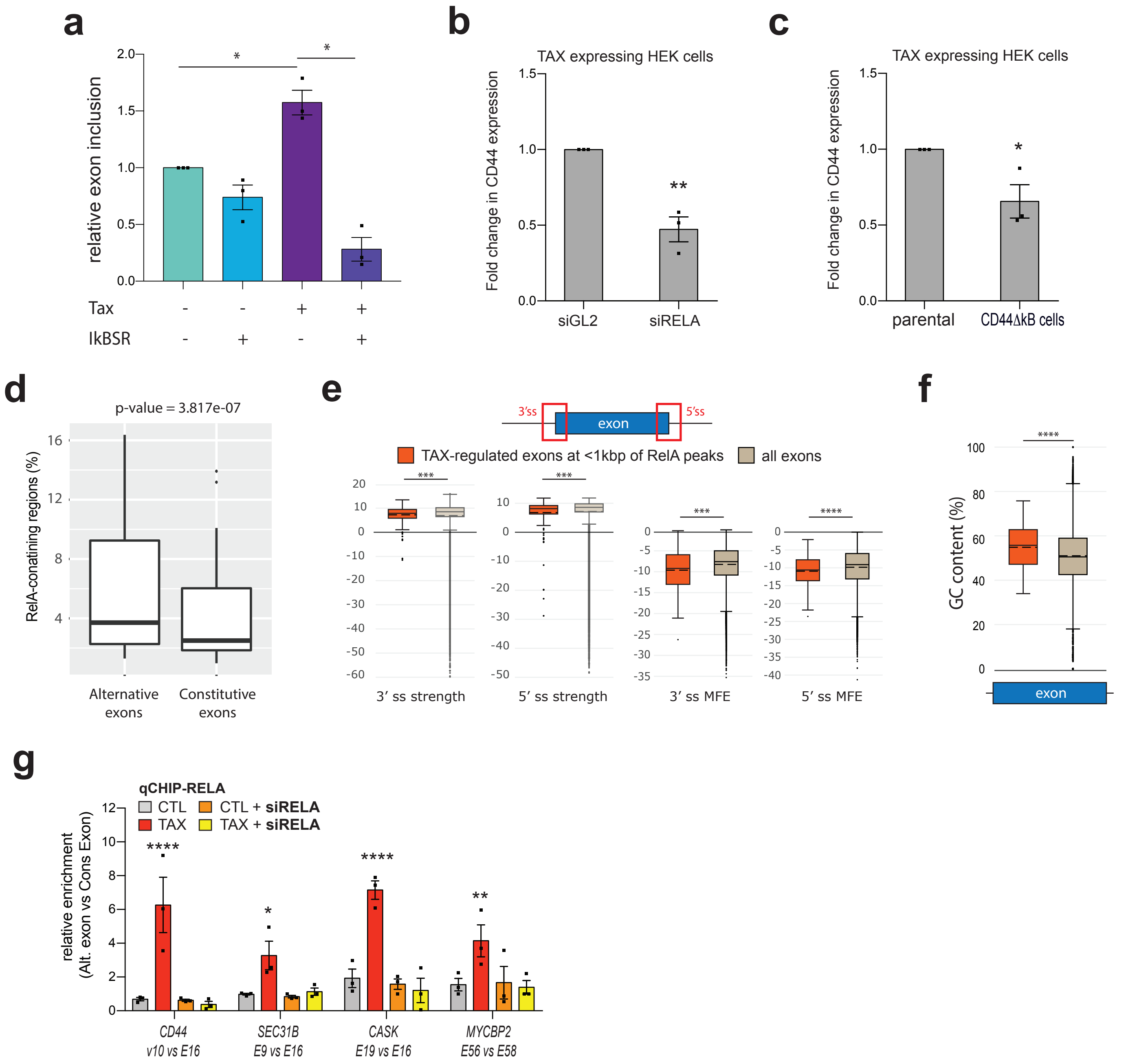

Supplementary Fig. 4: RELA occupancy and sequence features of Tax splicing targets.

a Tax-induced exon inclusion of CD44 exon v10 relies on NF-kB activation. HEK cells (293T-LTR-GFP) were transiently transfected with the Tax vector along with the IkBSR expression vector and the corresponding empty control vectors. The inclusion rate of exon v10 was quantified by qRT-PCR. **b** Relative fold-change in gene expression of CD44 upon siRNA-mediated silencing of RELA in Tax expressing HEK cells (Supplementary Fig. 2d). **c** Relative fold-change in gene expression of CD44 in CD44ΔkB HEK cells upon Tax expression. **d** Distribution of constitutive and alternative exons in RELA-enriched intragenic regions. ChIP-seq datasets were analyzed as detailed in method section. The groups "Constitutive exons" and "Alternative exons" contained 41873 and 103000 exons, respectively. The window was fixed to 3 kb upstream and downstream of each exon coordinates. Boxes extend from the 25th to 75th percentiles, the mid line represents the median and the whiskers indicate the maximum and the minimum values. **e** Tukey boxplots of strength and minimum free energy (MFE) of 5' and 3' splice sites, and GC content (**f**) of Tax-regulated exons localized at < 1 kbp of RELA binding site. The exons of FasterDB database were used as control. **g** RELA enrichment of Tax-regulated exons in cells expressing or not Tax and invalidated or not for RELA expression. Data in **a**, **b**, **c** and **g** are presented as the mean ± SEM values from biological replicates. Each black square represents a biological replicate. Statistical significance was determined with One-way ANOVA followed by Fisher's LSD test (**a** and **g**), two-tailed unpaired t-test (**b** and **c**), and Wilcoxon-Mann-Whitney test (**d, e,** and **f**) (*$p < 0.05$, **$p < 0.01$, ***$p < 0.001$, ****$p < 0.0001$). Source data are provided as a Source Data file.

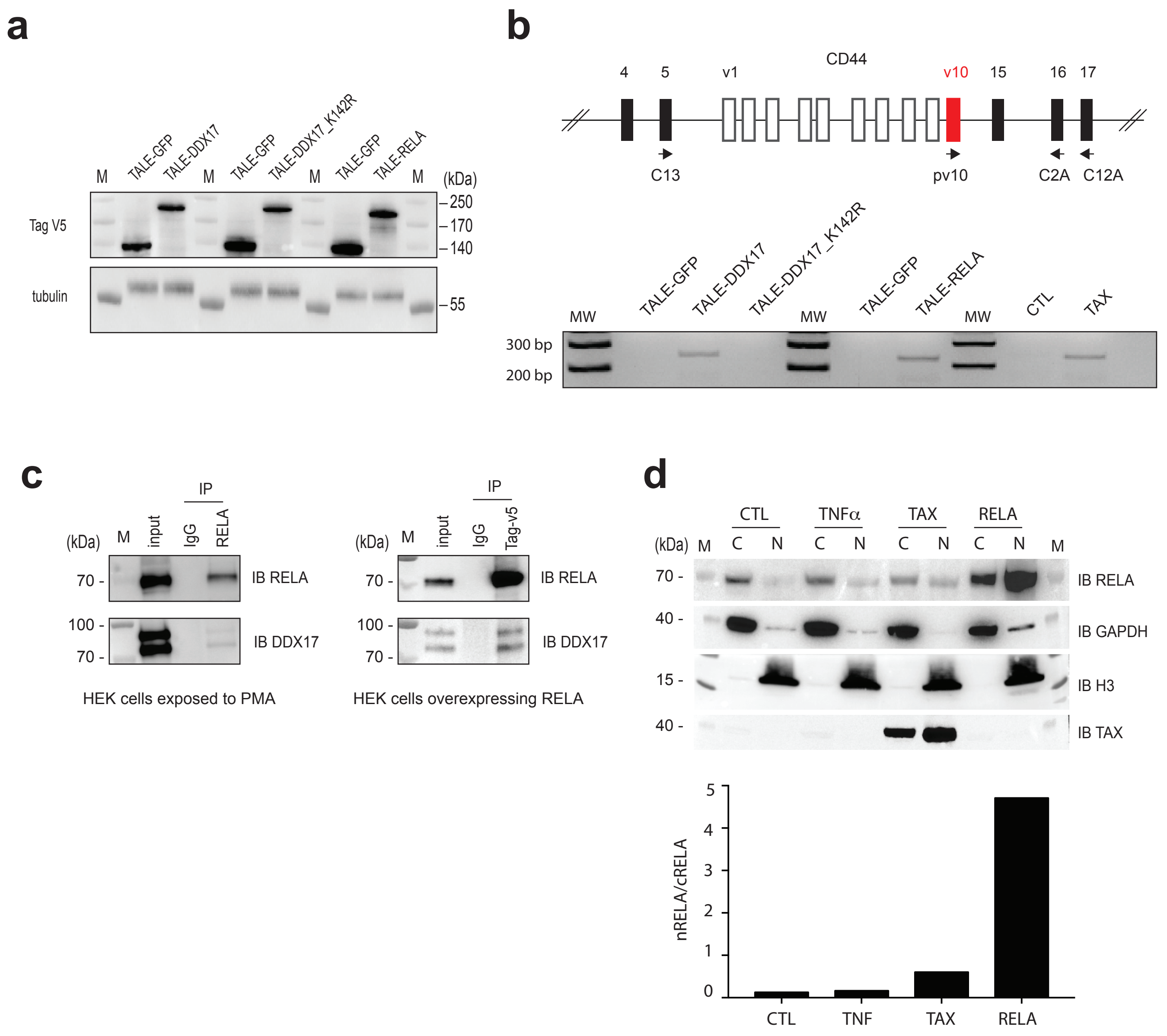

Supplementary Fig. 5: RELA/DDX17 chromatin interplay and splicing regulations.

a Western blot analysis of TALE-protein constructs in HEK cells using the anti-V5 Tag antibody. **b** Nested RT-PCR analysis of CD44 transcripts expressed in TALE assays and in cells expressing or not Tax. The first round of amplification consisted in 15 cycles of PCR with the primers C13 and C12A, the second round consisted in 35 cycles with primers pv10 and C2A. Final PCR products were resolved on a 1% agarose gel. **c** Co-immunoprecipitation assays of DDX17 and RELA using HEK cells exposed to PMA or expressing ectopic RELA. **d** subcellular fractionation and Western blotting were performed to assess the re-distribution of RELA in HEK cells upon TNFα exposure, TAX expression, or ectopic RELA expression using the expression vector pXJ41-v5-RELA. Cytosol (C) and nucleus (N) fractions are shown. GAPDH and Histone H3 were used as cytoplasmic and nuclear protein controls, respectively. nRELA/cRELA represents the ratio of nuclear RELA to cytoplasmic RELA. Representative image from three independent experiments is shown. Source data are provided as a Source Data file.

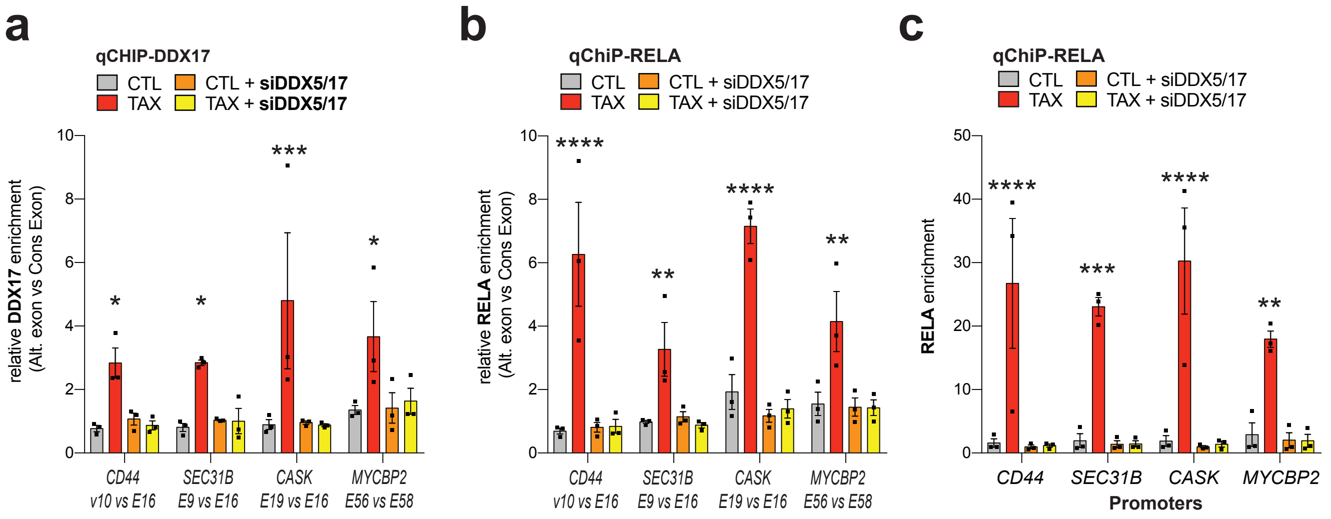

Supplementary Fig. 6: Impact of DDX5/17 depletion on RELA chromatin occupancy of exons and promoters regulated by Tax.
a Relative DDX17 occupancy of genomic exons regulated by Tax in HEK cells expressing or not Tax and treated or not with DDX5/17-specific siRNA. **b** Relative RELA occupancy at Tax-regulated exons in cells expressing or not Tax treated or not with DDX5/17-specific siRNA. **c** Relative RELA occupancy of promoters of Tax-regulated genes in cells expressing or not Tax and treated or not with DDX5/17-specific siRNA. Data are presented as the mean ± SEM values from biological replicates. Each black square represents a biological replicate. Statistical significance was determined with two-way ANOVA followed by Fisher's LSD test (*p < 0.05, **p < 0.01, ***p < 0.001, ****p < 0.0001). Exact pvalues (TAX vs CTL) are (**a**) 0.0308 (CD44), 0.0333 (SEC31B), 0.00016 (CASK), 0.016907 (MYCBP2) ; (**b**) <0.0001 (CD44), 0.0091 (SEC31B), <0.0001 (CASK), 0.0035 (MYCBP2), (**c**) <0.0001 (CD44), 0.0001 (SEC31B), <0.0001 (CASK), and 0.0039 (MYCBP2). Source data are provided as a Source Data file.
