# Supplementary material for: Intragenic recruitment of NF-κB drives splicing modifications upon activation by the oncogene Tax of HTLV-1
Source: Nat Commun. 2020 Jun 16;11:3045. doi: 10.1038/s41467-020-16853-x (PMC7298006; doi:10.1038/s41467-020-16853-x)
Supplement: Supplementary file 3 — Reporting Summary [file 41467_2020_16853_MOESM3_ESM.pdf]

nature research

Corresponding author(s): franck mortreux

Last updated by author(s): May 1, 2020

Reporting Summary

Nature Research wishes to improve the reproducibility of the work that we publish. This form provides structure for consistency and transparency in reporting. For further information on Nature Research policies, see our Editorial Policies and the Editorial Policy Checklist.

Statistics

For all statistical analyses, confirm that the following items are present in the figure legend, table legend, main text, or Methods section.

n/a	Confirmed	
☐	☒	The exact sample size (*n*) for each experimental group/condition, given as a discrete number and unit of measurement
☐	☒	A statement on whether measurements were taken from distinct samples or whether the same sample was measured repeatedly
☐	☒	The statistical test(s) used AND whether they are one- or two-sided<br>*Only common tests should be described solely by name; describe more complex techniques in the Methods section.*
☒	☐	A description of all covariates tested
☒	☐	A description of any assumptions or corrections, such as tests of normality and adjustment for multiple comparisons
☐	☒	A full description of the statistical parameters including central tendency (e.g. means) or other basic estimates (e.g. regression coefficient) AND variation (e.g. standard deviation) or associated estimates of uncertainty (e.g. confidence intervals)
☐	☒	For null hypothesis testing, the test statistic (e.g. *F*, *t*, *r*) with confidence intervals, effect sizes, degrees of freedom and *P* value noted<br>*Give P values as exact values whenever suitable.*
☒	☐	For Bayesian analysis, information on the choice of priors and Markov chain Monte Carlo settings
☒	☐	For hierarchical and complex designs, identification of the appropriate level for tests and full reporting of outcomes
☒	☐	Estimates of effect sizes (e.g. Cohen's *d*, Pearson's *r*), indicating how they were calculated

Our web collection on statistics for biologists contains articles on many of the points above.

Software and code

Policy information about availability of computer code

Data collection	Illumina HiSeq plateform (RNA-seq)
Data analysis	R Studio version 1.1.463<br>GraphPad Prism 7.0a (GraphPad Software Inc.)<br>CellQuest software (Becton Dickinson)<br>4200 TapeStation System (Agilent Technologies Inc.)<br>Light Cycler480 Software 5.1 (Roche Diagnostics GmbH)<br>Rotor-Gene 3000 cycler (Corbett)<br>Microsoft Office 2019 (Microsoft)<br>NanoDrop™ 2000 (Thermo Fisher Scientific)<br>ChemiDoc Touch Imaging System (BioRad)<br>Gel Doc XR+ System (BioRad)<br>ImageLab 6.0.1 (Bio-Rad)<br>CRISPRseek R package<br>FasterDB database (http://fasterdb.ens-lyon.fr)<br>FaRLine (https://omictools.com/farline-tool)<br>ggplot2 (v3.2.1)<br>Bowtie2 (v2.2.9)<br>HTSeq-count (v0.7.2)<br>DESeq2 (v1.10.1)<br>Macs2 (v2.1.1)<br>TRANSFAC v8.3

MEME-ChIP suite
MaxEntScan
RNAFold from ViennaRNA package (v 2.4.1)
Exon Ontology v1.5.0 (http://fasterdb.ens-lyon.fr/ExonOntology/)
DAVID software (http://david.abcc.ncifcrf.gov)

For manuscripts utilizing custom algorithms or software that are central to the research but not yet described in published literature, software must be made available to editors and reviewers. We strongly encourage code deposition in a community repository (e.g. GitHub). See the Nature Research guidelines for submitting code & software for further information.

Data

Policy information about availability of data

All manuscripts must include a data availability statement. This statement should provide the following information, where applicable:

- Accession codes, unique identifiers, or web links for publicly available datasets
- A list of figures that have associated raw data
- A description of any restrictions on data availability

RNA-seq data have been deposited on NCBI GEO under the accession number GSE123752.
The data EGAS00001001296 were generated by Department of Pathology and Tumor Biology, Kyoto University (https://www.ebi.ac.uk/ega/studies/EGAS00001001296).
Publicly available ChiP-seq datasets from GEO, ENCODE, and CISTROME databases are :
https://www.ncbi.nlm.nih.gov/geo/query/acc.cgi?acc=GSE63736
https://www.ncbi.nlm.nih.gov/geo/query/acc.cgi?acc=GSM1239484
https://www.ncbi.nlm.nih.gov/geo/query/acc.cgi?acc=GSM486271
https://www.ncbi.nlm.nih.gov/geo/query/acc.cgi?acc=GSM486293
https://www.ncbi.nlm.nih.gov/geo/query/acc.cgi?acc=GSM486298
https://www.ncbi.nlm.nih.gov/geo/query/acc.cgi?acc=GSM486318
https://www.ncbi.nlm.nih.gov/geo/query/acc.cgi?acc=GSE34329
https://www.ncbi.nlm.nih.gov/geo/query/acc.cgi?acc=GSM847877
https://www.ncbi.nlm.nih.gov/geo/query/acc.cgi?acc=GSM2394419
https://www.ncbi.nlm.nih.gov/geo/query/acc.cgi?acc=GSM2394421
https://www.ncbi.nlm.nih.gov/geo/query/acc.cgi?acc=GSM2394423
https://www.ncbi.nlm.nih.gov/geo/query/acc.cgi?acc=GSM2628088
https://www.encodeproject.org/files/ENCFF002CPA/
https://www.encodeproject.org/files/ENCFF002CQB/
https://www.encodeproject.org/files/ENCFF002CQJ/
https://www.encodeproject.org/files/ENCFF002CQN/
https://www.encodeproject.org/files/ENCFF580QGA/
http://cistrome.org/db/#/ for datasets 53597, 5388, 5389, 4940, 36310, 36316, and 4971.

The source data underlying Figs 1c, 2b-f, 3a-b, 3d, 4a, 4c-f, 5a-d, 6a-f, and Supplementary Figs 1, 2b-d, 3a, 3b, 3e, 4a-g, 5a-d, and 6a-c are provided as a Source Data file. All data supporting the findings of this study are available within the article and its supplementary information files and from the corresponding author upon reasonable request.

Field-specific reporting

Please select the one below that is the best fit for your research. If you are not sure, read the appropriate sections before making your selection.

- [x] Life sciences
- [ ] Behavioural & social sciences
- [ ] Ecological, evolutionary & environmental sciences

For a reference copy of the document with all sections, see nature.com/documents/nr-reporting-summary-flat.pdf

Life sciences study design

All studies must disclose on these points even when the disclosure is negative.

Sample size	Sample sizes are clearly stated in the figure legends and methods section.
Data exclusions	No data exclusion
Replication	All experiments were carried out in triplicate in order to verify the robustness of the data. All attempts for replication were successful.
Randomization	HEK cells, HTLV-1 tranformed cells, and MOLT4 cells were divided equally to each group and then treated with expression vectors, siRNA, and/or drugs. HTLV-1-infected and non-infected T-cell clones were randomly choosen amongst clones obtained by limiting dilution cloning of PBMCs.

Blinding	Blinding is not relevant in this study. However, the results were reproduced independently by at least two different authors.

Reporting for specific materials, systems and methods

We require information from authors about some types of materials, experimental systems and methods used in many studies. Here, indicate whether each material, system or method listed is relevant to your study. If you are not sure if a list item applies to your research, read the appropriate section before selecting a response.

Materials & experimental systems

n/a	Involved in the study
☐	☒ Antibodies
☐	☒ Eukaryotic cell lines
☒	☐ Palaeontology and archaeology
☒	☐ Animals and other organisms
☐	☒ Human research participants
☒	☐ Clinical data
☒	☐ Dual use research of concern

Methods

n/a	Involved in the study
☒	☐ ChIP-seq
☒	☐ Flow cytometry
☒	☐ MRI-based neuroimaging

Antibodies

Antibodies used

Primary antibodies : RELA (sc-109, Santa Cruz), Tax (1A3, Covalab), DDX17 (ProteinTech), DDX5 (ab10261 Abcam), Actin (sc-1616, SantaCruz), H3 (ab1791 Abcam), Alpha- tubulin (DM1A Santa CRUZ), V5 (AB3792, Millipore).
Secondary antibodies : Donkey anti-Goat IgG-heavy and light chain Antibody HRP Conjugated A50-101P Bethyl, Donkey anti-Rabbit IgG-heavy and light chain Antibody HRP Conjugated A120-108P Bethyl, Donkey anti-Mouse IgG-heavy and light chain Antibody HRP Conjugated A90-142P Bethyl, Mouse Anti-Rabbit IgG, light chain specific 211-032-171 Jackson ImmunoResearch and Goat Anti-Mouse IgG, light chain specific 115-035-174 Jackson ImmunoResearch

Validation

All antibodies were used as specified and validated by the manufacturer. All signals in Western blot analysis were consistent with the predicted size of target protein. For qChip analysis, positive and negative targets validated the specific enrichments.

Validation information for each antibody can be found in manufacturer's website as listed below:
Anti-RELA (sc-109), Santa Cruz : http://datasheets.scbt.com/sc-109.pdf
Anti-NFκBp65 (C-20) sc-372, Santa Cruz : https://datasheets.scbt.com/sc-372.pdf
Anti-HTLV-1 Tax (1A3), Covalab: https://www.covalab.com/htlv-1-tax-antibody-1a3-1.html
Anti-DDX17 (ab24601), Abcam: https://www.abcam.com/ddx17-antibody-ab24601.html
Anti-DDX17 19910-1-AP, Proteintech : https://www.ptglab.com/Products/DDX17,P72-Antibody-19910-1-AP.htm
Anti-DDX5 (ab10261), Abcam: https://www.abcam.com/ddx5-antibody-ab10261.html
Anti-Actin(I-19):sc-1616, Santa Cruz: https://datasheets.scbt.com/sc-1616.pdf
Anti-V5 Epitope Tag Antibody (AB3792), Millipore: https://www.merckmillipore.com/FR/fr/product/Anti-V5-Epitope-Tag-Antibody,MM_NF-AB3792
Anti-GAPDH (6C5) sc-32233, Santa Cruz : https://datasheets.scbt.com/sc-32233.pdf
Anti-Histone H3 antibody - Nuclear Loading Control and ChIP Grade (ab1791), Abcam: https://www.abcam.com/histone-h3-antibody-nuclear-loading-control-and-chip-grade-ab1791.html
Anti-α Tubulin (DM1A) sc-32293, Santa Cruz: https://datasheets.scbt.com/sc-32293.pdf
Donkey anti-Goat IgG-heavy and light chain Antibody HRP Conjugated A50-101P Bethyl:
https://www.bethyl.com/product/pdf/A50-101P.pdf
Donkey anti-Rabbit IgG-heavy and light chain Antibody HRP Conjugated A120-108P Bethyl:
https://www.bethyl.com/product/pdf/A120-108P.pdf
Donkey anti-Mouse IgG-heavy and light chain Antibody HRP Conjugated A90-142P Bethyl:
https://www.bethyl.com/product/pdf/A90-142P.pdf
Mouse Anti-Rabbit IgG, light chain specific 211-032-171, Jackson ImmunoResearch:
https://www.jacksonimmuno.com/catalog/products/211-032-171
Goat Anti-Mouse IgG, light chain specific 115-035-174, Jackson ImmunoResearch:
https://www.jacksonimmuno.com/catalog/products/115-035-174

Eukaryotic cell lines

Policy information about cell lines

Cell line source(s)

The human embryonic kidney 293T-LTR-GFP cells have been previously described by Delebecque et al., J Virol 2002 and they were provided by Frederic Tangy (Pasteur Institute, Paris, France). HTLV-1 infected cell lines ATL2 and C91PL have been previously described by K Ikuta et al., PNAS 1985 and M Popovic, PNAS, 1983, respectively. ATL-2 cells were provided by Masao Matsuoka (Kyoto University, Kyoto, Japan) and Roberto Accolla (Università degli Studi dell'Insubria, Varese, Italia)), C91PL were provided by Cynthia Pise-Masison (National Cancer Institute, NIH, Bethesda, MD) and Renaud Mahieux (Center for Research in Infectious Diseases, Lyon, France). The non-infected MOLT4 cells come from ATCC (CRL-1582).

Authentication	None of the cell lines used were authenticated.
Mycoplasma contamination	cell lines were tested for mycoplasm contamination and all were negative.
Commonly misidentified lines (See ICLAC register)	no commonly misidentified cell lines were used in the study.

Human research participants

Policy information about studies involving human research participants

Population characteristics	CD4+ clones were obtained through cloning by limiting dilution of peripheral blood mononuclear cells (PBMCs) derived from 2 HTLV-1-infected individuals.
Recruitment	Participants were recruited based on seropositivity for HTLV-1.
Ethics oversight	the biomedical research program was approved by the Committee for the Protection of Persons, Ile-de-France II, Paris (2012-10-04 SC). All patients provided writen informed consent.

Note that full information on the approval of the study protocol must also be provided in the manuscript.
