# Supplementary material for: Intragenic recruitment of NF-κB drives splicing modifications upon activation by the oncogene Tax of HTLV-1
Source: Nat Commun. 2020 Jun 16;11:3045. doi: 10.1038/s41467-020-16853-x (PMC7298006; doi:10.1038/s41467-020-16853-x)
Supplement: Supplementary file 4 — Description of Additional Supplementary Files [file 41467_2020_16853_MOESM4_ESM.pdf]

Description of Additional Supplementary Files

Supplementary Data 1: Whole gene expression and alternative splicing changes upon Tax expression. Whole gene expression threshold was set to log2(FC) = 0.6, $p < 0.05$ (glm and Wald test). For differential splicing, deltaPsi threshold was set to 1.1 p<0.05 (Fisher test). Tax-induced splicing regulation identified in RNA-seq datasets derived from carriers and ATLL samples (EGAS00001001296). Alternative splicing profiles of each clinical sample was assessed using Farline analysis, with peripheral blood CD4+ T-cells used as a control. The table lists 452 Tax-induced splicing regulations identified at least once across 56 clinical samples. The sheet “samples EGAS00001001296” lists primary cell samples published by Kataoka and colleagues[24]. The sheet “PSI_donors_carriers_ATLL” presents the psi values for exons expressed in donors, asymptomatic carriers, and ATL patients.

Supplementary Data 2: Sequence features of exons regulated by Tax.

Supplementary Data 3: Genes modified in splicing by Tax in a DDX5/17-dependent manner. Tax splicing targets responsive to DDX5/17 were attributed to splicing events lost upon DDX5/17 depletion.

Supplementary Data 4: Protein features encoded by Tax-regulated exons.

Supplementary Data 5: List of oligonucleotides, siRNAs, sgRNAs, and TALE sequences.
